# Occupational stress and mental suffering among emergency staff

**DOI:** 10.1192/j.eurpsy.2021.1916

**Published:** 2021-08-13

**Authors:** F. Dhouib, N. Kotti, R. Masmoudi, A. Hrairi, K. Jmal Hammami, M. Larbi Masmoudi, J. Masmoudi, M. Hajjeji

**Affiliations:** 1 Department Of Occupational Medicine, HEDI CHAKER hospital, SFAX, Tunisia; 2 Psychiatry A, hedi chaker hospital, Sfax, Tunisia; 3 The Department Of Occupational Medicine And Work-related Pathology, University hospital Hedi Chaker Sfax, SFAX, Tunisia; 4 Psychiatrie “a” Department, Hedi Chaker Hospital University -Sfax - Tunisia, sfax, Tunisia

**Keywords:** Work stress, psychic suffering, emergency staff, violence

## Abstract

**Introduction:**

In emergency departments, workers regularly report unfavorable working conditions. It’s a stressful workplace with excessive workloads, high demands on patient care including exposure to violence, time pressures and overcrowding.

**Objectives:**

-Identify the prevalence of violence, psychological distress and job stress in the emergency department. -Investigate risk factors of aggression among emergency department staff.

**Methods:**

This study is a descriptive-cross sectional analysis study conducted on emergency department staff. Data were collected through a questionnaire including sociodemographic information, circumstances of the aggression, the Karasek questionnaire, and the GHQ-12 (General Health Questionnaire).

**Results:**

Our study included 62 health workers. Half of the population were physicians. The prevalence of assault was estimated at 59.6%. Psychological distress was noted in 64.5% of cases. Occupational stress was estimated at 75.8%. The most common type of assault was verbal aggression (86.4%). The aggressor was most often an accompanying person. The occurrence of violence was not associated with the worker’s psychological distress or job stress. On the other hand, we noted an association between aggression and variable work schedule.

**Conclusions:**

Violence against healthcare workers in the emergency department is an important phenomenon. Preventive actions should be taken to improve health professional wellbeing at work.

**Disclosure:**

No significant relationships.

